# Case Report: Bilateral lens dislocation as an atypical presentation of acromegaly and review of the ocular effects of GH/IGF-1 excess

**DOI:** 10.3389/fendo.2025.1666425

**Published:** 2025-10-27

**Authors:** Laura Vitale, Letizia Maria Fatti, Marco Bonomi, Stefano Frara, Giovanni Vitale, Luca Persani, Biagio Cangiano

**Affiliations:** ^1^ Dept of Medical Biotechnology and Translational Medicine, University of Milan, Milan, Italy; ^2^ Istituto Auxologico Italiano IRCCS, Division of Endocrine and Metabolic Diseases, Milan, Italy; ^3^ Department of Life Science, Helath and health Professions, Università degli Studi Link, Rome, Italy

**Keywords:** acromegaly, pasireotide, diabetes, lens dislocation, complication, Eye manifestations, Insulin-like growth factor I

## Abstract

**Introduction:**

We report the case of a 71-year-old woman with acromegalic facies, referred following bilateral idiopathic lens luxation (LL). Subsequent investigations revealed a 15-mm pituitary adenoma, along with biochemical evidence of massive growth hormone hypersecretion (Growth Hormone (GH): 93.22 µg/L; insulin-like growth factor 1 [IGF-1]: 748 µg/L), consistent with acromegaly. She exhibited multiple comorbidities, including arterial hypertension, chronic heart failure secondary to dilated cardiomyopathy—compatible with acromegalic heart disease (AHD)—osteoporosis, and type 2 diabetes mellitus (T2DM), reflecting a long-standing and high-burden disease.

**Treatment and clinical course:**

Since the patient was not eligible for surgery and daily subcutaneous injections were unfeasible due to the patient’s lack of autonomy and limited caregiver support, therapy with lanreotide was initiated despite complete resistance to high doses of the classic analogue. We switched to pasireotide, achieving excellent disease control with 60 mg administered every 28 days. Progressive reduction in IGF-1 levels subsequently allowed a dose tapering to 40 mg every 28 days. Biochemical control of acromegaly was accompanied by improvement in disease-related complications (most notably T2DM), as well as the development of secondary hypocortisolism.

**Ocular complications:**

Bilateral lens dislocation is not a known acromegaly complication; however, its bilateral occurrence suggests an underlying systemic cause. A plausible pathogenetic mechanism may involve chronic GH hypersecretion and IGF-1 overexpression, with subsequent interaction with ocular receptors. IGF-1 exerts an antiapoptotic and pro-proliferative action on lenticular cells through interaction with the IGF-1 receptor and the intracellular PI3K/Akt pathway. It is a regulatory factor in the synthesis and degradation of fibrillin-1, a glycoprotein abundantly expressed in the extracellular matrix of the ciliary zonule, whose altered synthesis may underlie weakness of the lens suspensory apparatus. This is the first reported case of its genre, although bilateral intraocular lens subluxation (LS) in a patient with acromegaly and elevated intraocular pressure has previously been reported.

**Conclusions:**

Bilateral lens dislocation may represent an atypical presentation of acromegaly. It may indicate advanced disease and, if confirmed in other cohorts, could be considered among the suggestive signs of acromegaly. In our case, the use of pasireotide allowed adherence to therapy and optimal therapeutic response in a multicomplicated, non-self-sufficient patient.

## Introduction

Acromegaly is a rare disease caused by hypersecretion of GH of pituitary or, in rare instances, of extra-pituitary origin. The disease is characterised by progressive somatic overgrowth involving bones, cartilage, and soft tissues, and is frequently associated with a broad spectrum of systemic comorbidities (1). Here, we report the case of a patient with acromegaly and a substantial disease burden, reflected by markedly elevated insulin-like growth factor 1 (IGF-1) levels and multiple severe comorbidities, who presented with spontaneous bilateral lens luxation (LL). To the best of our knowledge, this is the first documented case of such an occurrence. A prior report in 2020 reported bilateral intraocular lens subluxation (LS) in a patient with acromegaly and elevated intraocular pressure. We propose that bilateral lens dislocation may represent an atypical presentation of acromegaly, especially in cases of advanced disease.

## Case description

In September 2021, a 71-year-old woman came to our clinic, as suggested by her ophthalmologist, following the recognition of acromegaloid features in the context of recent idiopathic bilateral lens dislocation. On examination, the patient exhibited macroglossia and frontal bossing. There was no family history of pituitary adenomas or other endocrine disorders. Her clinical profile was notable for multiple comorbidities commonly associated with acromegaly, including: essential hypertension, for which she was on antihypertensive medication; chronic heart failure secondary to dilated cardiomyopathy (consistent with AHD), managed with diuretics; obstructive sleep apnoea syndrome (OSAS) coupled with chronic pneumopathy due to severe kyphoscoliosis; osteoporosis with a recent low-energy femoral fracture; and type 2 diabetes mellitus (T2DM), managed with basal insulin and the glucagon-like peptide-1 receptor agonist liraglutide. Additional medical history included intellectual disability (oligophrenia), reduced mobility, and a prior colectomy with permanent colostomy following volvulus-induced bowel obstruction.

## Diagnostic assessment

Biochemical evaluation revealed a massive hypersecretion of GH and IGF-1 (GH: 93.22 µg/L [reference range: < 4.00], IGF-1: 748 µg/L [reference range: 51–176]). Associated findings included hypogonadotropic hypogonadism and a cortisol level in the intermediate cortisol level, prompting dynamic testing. An adrenocorticotropic hormone (ACTH) stimulation test subsequently confirmed preserved adrenal function.

Neuroimaging with computed tomography—performed instead of magnetic resonance imaging (MRI) due to the patient’s severe kyphoscoliosis—revealed a 15-mm expansive lesion in the sellar area, which appeared slightly hyperdense on baseline imaging, with homogeneous contrast enhancement.

The diagnosis of acromegaly was confirmed, defined by IGF-1 > 1.3 times the upper limit of normal for age in a patient with typical signs and symptoms of acromegaly (2).

Follow-up of known acromegalic complications and screening for previously undiagnosed ones was initiated, with **Table 1** reporting the most clinically relevant findings.

**Table 1:** Most significant patient comorbidities and their possible relationships with acromegaly.

Diagnosis/system affected	Possible relationship to acromegaly
Systemic arterial hypertension/cardiovascular system	Cardiovascular disease represents the most prevalent and often not remissible comorbidity in patients with acromegaly, accounting for up to 80% of acromegalic complications (3). Up to two-thirds of patients suffer from arterial hypertension (4). Higher risk of hypertension probably results from concomitant factors leading to expansion of extracellular fluid volume, increased peripheral vascular resistance, and development of sleep apnoea syndrome (5).
Bilateral carotid atheromasia/arteries	Compared with the healthy population, patients with acromegaly have a higher prevalence, variable based on case history, of endothelial dysfunction and carotid atheromasia (6), due to the presence of multiple cardiovascular risk factors such as hypertension, an atherogenic lipid profile, insulin resistance, and diabetes mellitus (7).
Acromegalic heart disease (AHD)/heart	Characterized by biventricular hypertrophy or increased interventricular septum thickness (eccentric hypertrophy), diastolic dysfunction at rest, and/or systolic dysfunction on effort (3, 8). The patient’s transthoracic echocardiogram documented a mild hypertrophy of the left ventricle, an initial diastolic decompensation with E/A 0.6, and a diastolic pattern due to altered relaxation with E/E’ 7.25.
Type 2 diabetes mellitus/glycaemic metabolism	Acromegaly is associated in about 30% (9) of cases with a particular type of diabetes mellitus, characterized paradoxically by a lean phenotype and reduced adipose tissue mass, in which insulin resistance arises independently of obesity due to lipolytic and insulin resistance-inducing effects of growth hormone (10, 11).
Osteoporosis complicated by femoral fracture following low-energy trauma and suspected vertebral fractures (due to severe kyphoscoliosis), with altered bone metabolism.	Acromegalic osteoporosis is characterized by a higher risk of fractures, a normal BMD due to increased bone turnover, and deterioration in bone microarchitecture, but coexisting increase in cortical and trabecular bone mass (12).
Low-grade primary renal neoformation/kidney	Coherent with higher risks of neoplasm development due to the pro-proliferative action of IGF-1 (13).

Regarding ocular involvement, in August 2021, the patient underwent a routine ophthalmologic examination as part of her annual diabetic retinopathy screening. During the visit, she reported a prior episode of sudden visual impairment. Due to her longstanding intellectual disability, she had not previously communicated any visual complaints; however, upon detailed questioning, she reported that the visual impairment had developed spontaneously within the preceding days to weeks. Slit-lamp examination revealed bilateral lens dislocation into the vitreous cavity. The patient was promptly referred to the nearest ophthalmologic emergency department for further evaluation and management. The LLs were addressed with the implantation of scleral-fixated intraocular lenses (IOLs); the right eye subsequently experienced a bulbar rupture, resulting in loss of function, while the left eye recovered without complications, preserving vision.

Given the idiopathic nature of the bilateral lens dislocation and the absence of trauma or other identifiable causes, the ophthalmologist referred the patient for endocrinological evaluation to investigate a possible underlying systemic etiopathogenesis.

## Therapeutic intervention

According to the 2025 Consensus, surgery remains the first-line treatment for acromegaly (14); however, our patient was not a candidate for surgical intervention due to her severe comorbidities and poor general health status. Current clinical guidelines emphasise a personalised approach to acromegaly management, considering factors such as patient preferences (e.g., oral vs. injectable formulations), comorbid conditions (e.g., type 2 diabetes mellitus), disease-specific features (e.g., MRI findings, degree of IGF-1 elevation), and the potential adverse effects of therapy (e.g., hyperglycaemia). Somatostatin receptor ligands (SRLs) are considered the cornerstone of medical therapy. However, pegvisomant monotherapy may be a valuable alternative in patients with significantly impaired glucose metabolism, no concerns regarding adenoma mass, and markedly elevated IGF-1 levels (14). In the present case, due to the patient’s lack of autonomy and limited caregiver support, daily injections were not feasible. Consequently, long-acting lanreotide was initiated at a dose of 90 mg every 28 days.

Lanreotide only slightly reduced GH levels while failing to control IGF-1 concentrations. In accordance with current guidelines, gradual dose escalations were implemented, reaching up to 120 mg every 25 days; however, therapy remained ineffective, indicating complete resistance to the drug.

In cases of suboptimal response to SRLs and a high risk of new-onset or worsening diabetes mellitus, current guidelines recommend considering pegvisomant monotherapy or the addition of cabergoline if disease control is only marginally insufficient (14). However, persistent marked IGF-1 hypersecretion and the patient’s inability to adhere to daily injections precluded both options. Pasireotide is recognised as a valuable treatment for patients with acromegaly who remain uncontrolled despite maximally titrated doses of first-generation SRLs, as demonstrated in the PAOLA study (2014) (15) and supported by a recent systematic review (16). Nevertheless, pasireotide administration requires careful monitoring due to its association with increased glycaemia, glycated hemoglobin (HbA1c) levels, and a higher incidence of T2DM; its use should be carefully monitored in patients with uncontrolled T2DM (17). Consequently, second-line therapy with pasireotide long-acting release (LAR) was initiated, with close monitoring of T2DM throughout follow-up. Treatment with this new-generation SRL at a dose of 60 mg every 28 days achieved excellent disease control. This effect is likely due to pasireotide’s greater affinity for somatostatin receptor subtype 5 (SSTR-5), which allowed it to overcome the resistance to classic SRL (18).

**Figure 1:**
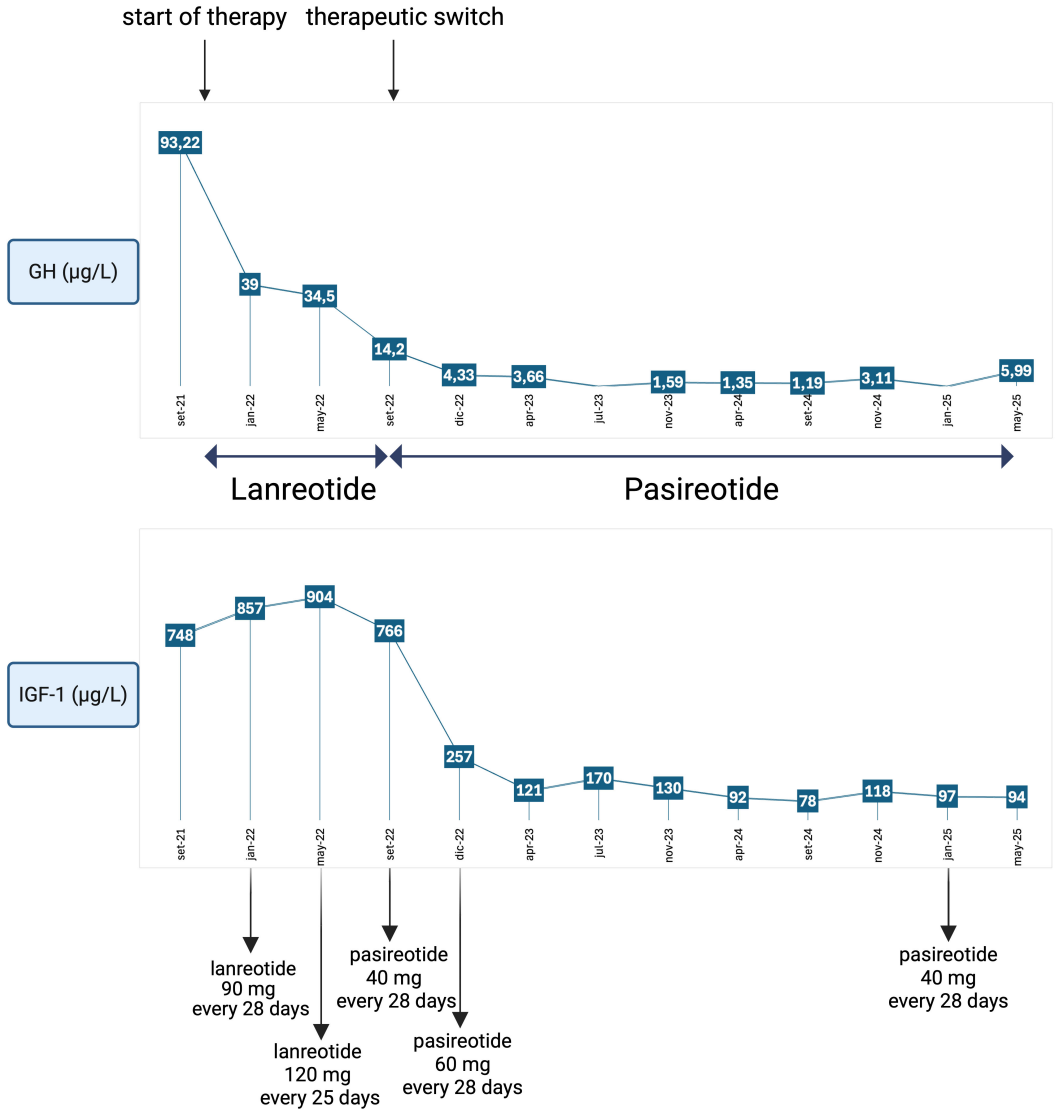
Trend of GH and IGF-1 values during therapy with a first-generation somatostatin analogue (lanreotide) at incremental dosages and with a new-generation somatostatin analogue (pasireotide), showing excellent disease control even after dosage reduction. Reference ranges: IGF-1: 51–176 µg/L; GH: < 4.00 µg/L. Created in BioRender. Vitale, L. (2025) https://BioRender.com/q1ke5xg.

IGF-1 levels progressively decreased, allowing concurrent reduction of pasireotide dosage to 40 mg (see **Figure 1**). Current consensus on criteria for acromegaly diagnosis and remission recommends assessing IGF-1 levels at 3 months after therapy initiation, followed by evaluations every 6–12 months after biochemical control is achieved. Biochemical control is defined as normalisation of IGF-1 concentrations within the age-adjusted reference range. Random GH evaluation is considered helpful only in cases of concern about adenoma behaviour (2). In this case, IGF-1 was monitored every 3–4 months, coinciding with the patient’s quarterly outpatient visits for in-hospital drug dispensing. This approach enabled closer clinical surveillance, considered necessary due to the patient’s overall frailty and complex clinical profile. Additionally, random GH levels were periodically measured to indirectly monitor pituitary adenoma activity, since imaging was not feasible.

Alongside monitoring biochemical disease control, follow-up also included assessment of acromegaly-related comorbidities and complications, in accordance with current clinical guidelines (2).

## Follow-up and outcome

**Figure 2:**
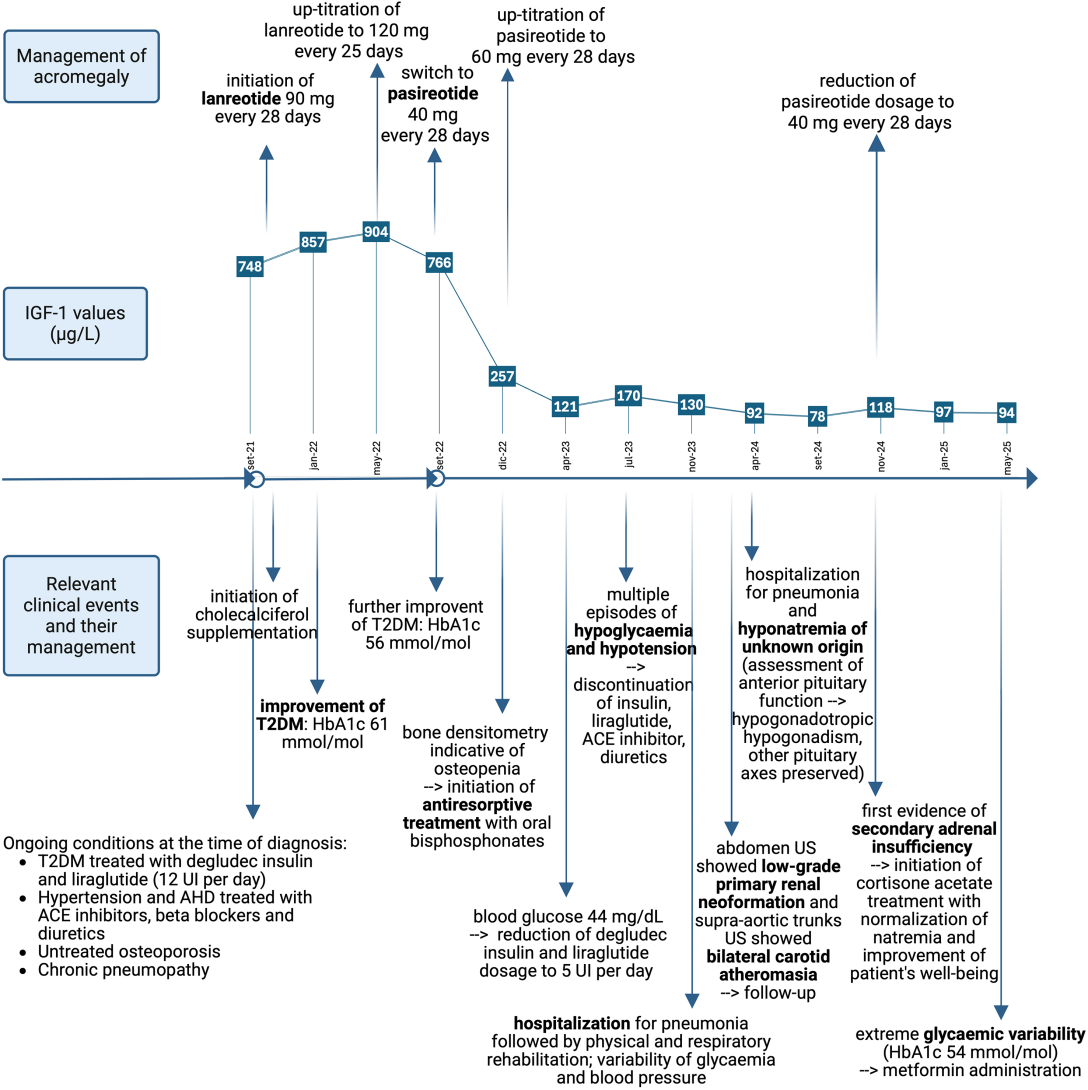
Timeline of key clinical events, their management, and concurrent IGF-1 values in the management of acromegaly. T2DM significantly improved during both lanreotide and pasireotide treatments. Cardiovascular conditions improved with the detection of hypotension, which led to suspension of diuretics and antihypertensive drugs. Variability of glycaemia and blood pressure persisted throughout the follow-up. Primary renal neoplasm and bilateral carotid atherosclerosis were detected, and follow-up was initiated. Hyponatremia and secondary adrenal insufficiency were detected, and replacement therapy was initiated. Osteoporosis was managed with antiresorptive treatment and cholecalciferol supplementation. Poor general health conditions led to hospitalisation due to pneumonia. T2DM, type 2 diabetes mellitus; AHD, acromegalic heart disease; HbA1c, glycated haemoglobin; US, ultrasound sonography. Reference ranges: IGF-1: 51–176 µg/L; HbA1c: < 48 mmol/mol. Created in BioRender. Vitale, L. (2025) https://BioRender.com/ten8nep.

The broad spectrum of acromegaly-related comorbidities reflects both a substantial disease burden and a long-term condition, which often limits the reversibility of comorbidities. Nevertheless, alongside reductions in GH and IGF-1 levels, several comorbidities showed significant improvement (see **Figure 2**).

Cardiovascular status improved to the extent that antihypertensive agents and diuretics were temporarily discontinued.

Antiresorptive therapy was administered without evidence of new fractures; however, the patient’s osteoporosis had already contributed to clinical frailty. Severe kyphoscoliosis—potentially exacerbated by suspected vertebral fractures (morphometric assessment was hardly performable)—led to hypomobility and chronic pulmonary disease, which required pulmonary and physical rehabilitation. Osteoporosis severity may be due to postmenopausal hypoestrogenism or hypopituitarism of uncertain onset (19), compounded by chronic GH/IGF-1 excess (12).

T2DM, diagnosed in 2019 and poorly controlled at acromegaly presentation (HbA1c: 85 mmol/mol; reference range: < 42 mmol/mol), improved significantly during lanreotide therapy (HbA1c: 56 mmol/mol). Pasireotide is known to induce or worsen T2DM in 57% (20)–63% (21) of patients due to its inhibitory effect on incretin secretion via SSTR-5 pancreatic cell receptors (22). Unexpectedly, with acromegaly control, blood glucose levels rapidly declined, allowing complete discontinuation of antidiabetic medications due to episodes of severe hypoglycaemia. This strongly suggests that the patient’s diabetes was secondary to acromegaly and GH hypersecretion. While T2DM improvement may be considered novel, pasireotide’s effectiveness in achieving disease control is well established (15, 16).

In this elderly, nonautonomous patient with multiple comorbidities, pasireotide offered several advantages, including ease of administration (one intramuscular injection every 28 days), rapid and sustained disease control, and reduction of disease burden with improvement in symptoms and complications, particularly T2DM.

Collaterally, central hypocortisolism was observed, and replacement therapy with cortisone acetate was initiated. This may be secondary to adenoma shrinkage or possible pituitary apoplexy; however, the absence of imaging data limits confirmation of this hypothesis.

## Discussion

Given that pretreatment IGF-1 levels are important predictors of morbidity and mortality in acromegaly (23), our patient not only experienced prolonged exposure to elevated IGF-1 and a high disease burden but was also ineligible for first-line therapy. This sustained hormonal excess likely contributed to the development of progressively severe complications, including ocular manifestations.

Multiple studies have identified various GH and IGF-1 target sites within ocular tissues, supporting an association between acromegaly and a spectrum of ocular abnormalities. These include increased iris thickness, reduced iris curvature, a wider anterior chamber angle (24), as well as increased corneal and lens thickness. Additionally, retinal changes such as thinning of the retinal nerve fibre layer, ganglion cell layer, and inner plexiform layer have been reported (25).

Furthermore, evidence indicates that GH—and particularly its effector IGF-1—acts on lens cells and their suspensory apparatus via IGF-1 receptor interactions. *In vitro* studies using bovine and chicken lens epithelial cells demonstrated expression of IGF-1 binding sites and the ability to synthesise and release IGF-1, potentially establishing a concentration gradient maintained by locally produced IGF-1 and by IGF-1 in the vitreous humour, which may reflect systemic GH and IGF-1 levels (26, 27).

Specifically, IGF-1 exerts antiapoptotic and pro-proliferative effects on lens epithelial cells through activation of the phosphatidylinositol 3-kinase (PI3K) pathway. Downstream signalling diverges into two branches: one mediates antiapoptotic effects via the PI3K/Protein Kinase B (Akt) pathway, which inactivates the pro-apoptotic protein Bad and suppresses caspase activation; the other promotes cell proliferation through stimulation of the PI3K/p70 ribosomal S6 kinase (p70 S6) kinase cascade (28). Additionally, while Zang et al. reported a nonstatistically significant increase in lens thickness in patients with acromegaly compared to controls, Batur et al. later confirmed this finding with statistical significance, supporting the role of GH and IGF-1 in lens thickening. However, correlation analyses revealed only weak associations between lens thickness and disease duration, IGF-1, or GH levels. Moreover, IGF-1 has been shown to stimulate fibroblast growth factor, inducing lens fibre differentiation in rat models (29).

Given its stimulatory effects on the proliferation and differentiation of lenticular cells, the same authors demonstrated that IGF-1 also acts on the ciliary zonule, the fibrillar structure connecting the lens to the ciliary body and maintaining lens positioning. IGF-1 serves as a key regulatory factor in the synthesis and degradation of fibrillin-1, a glycoprotein abundantly expressed in the extracellular matrix of the ciliary zonule. This regulatory role is mediated through the interaction between the IGF-1 receptor and the PI3K/Akt pathway, as well as mTOR and p70 S6 kinase signalling (30), with studies showing that inhibition of this pathway impairs fibrillin-1 synthesis. However, several factors regulate the synthesis and degradation of fibrillin-1, whose altered production can weaken the lens suspensory apparatus; nonetheless, IGF-1 is likely a major contributor to this process.

**Figure 3:**
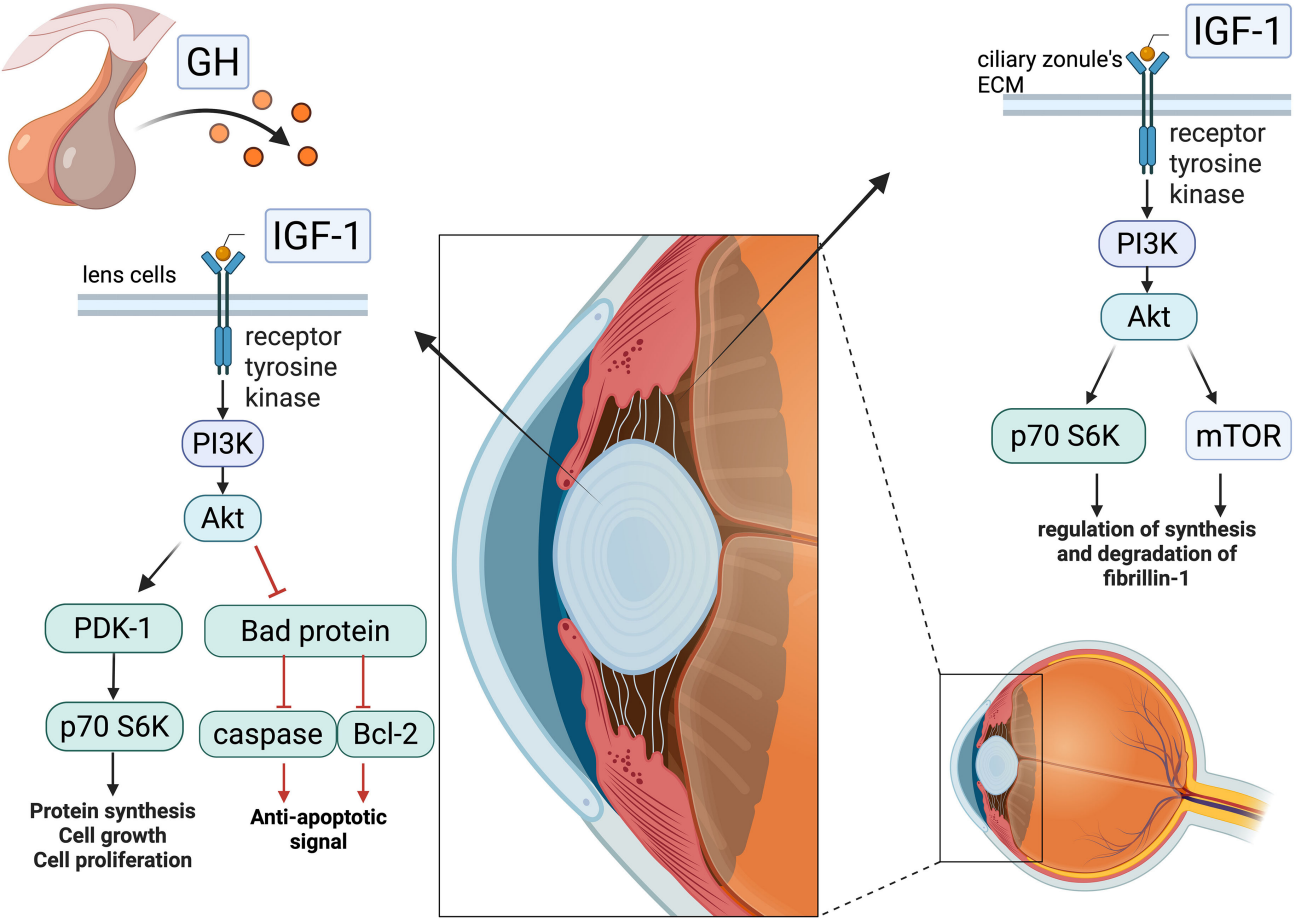
Effect of IGF-1’s interaction with cells of the lens and ciliary body: intracellular IGF-1 pathway—following interaction with the tyrosine kinase receptor, IGF-1 initiates a phosphorylation cascade that bifurcates, in lenticular cells, into a pro-proliferative signal and an antiapoptotic one, while in the ciliary zonule, it regulates the synthesis and degradation of fibrillin-1. In the presence of high IGF-1 concentrations, these events may contribute to the weakening of the lens suspensory apparatus and lead to increased lens thickness, promoting its dislocation. Created in BioRender. Vitale, l. (2025) https://BioRender.com/ez3w9xi.

In particular, chronic exposure to elevated levels of GH and IGF-1—as seen in patients with a significant disease burden, characterised by persistently high hormone levels and multiple comorbidities indicative of longstanding disease—may contribute to ocular alterations such as lens thickening and dysfunction of the lens suspensory apparatus, potentially increasing the risk of LL (see **Figure 3**).

To the best of our knowledge, this is the first case of its kind reported to date. In 2020, Chang and Chen reported a case of late bilateral IOL subluxation in a patient with acromegaly. The patient had previously undergone IOL implantation for cataract correction and later developed elevated intraocular pressure—a known ocular complication of acromegaly—which was treated and controlled. However, subsequent slit-lamp examination revealed bilateral inferior subluxation of the IOLs. The authors attributed this finding to weakening of the IOL suspensory apparatus, likely resulting from IGF-1-mediated effects on myofibrils and the extracellular matrix (ECM) of the zonular fibres. Additionally, they proposed a potential role for matrix metalloproteinases, which are highly expressed in pituitary adenomas and may release growth factors anchored to the ECM, potentially degrading fibrillin-1 molecules and further contributing to ciliary apparatus weakness (31).

Bilateral LL may represent an atypical presentation of acromegaly. If confirmed in larger cohorts, it could indicate advanced disease and potentially be included among the suggestive signs of acromegaly.

## Patient perspective

In this case, treatment with pasireotide facilitated adherence to therapy and achieved optimal disease control in a complex, nonautonomous patient with limited caregiver support. The patient also experienced significant improvement in disease symptoms and related complications.

## Data Availability

The original contributions presented in the study are included in the article/supplementary material. Further inquiries can be directed to the corresponding author.
